# Supplementary material for: Development of Traceable Mouse Models of Advanced and Metastatic Bladder Cancer
Source: Cancers (Basel). 2024 Jun 17;16(12):2245. doi: 10.3390/cancers16122245 (PMC11202260; doi:10.3390/cancers16122245)

cancers MDPI

Supplementary Materials:

Figure S1. Adenovirus-induced Cre-Lox deletions. In $p53^{fl/fl}pten^{fl/fl}Luc\text{-}SIY^{-}$ and $p53^{fl/fl}pten^{fl/fl}Luc\text{-}SIY^{+}$ mice, the floxed *tp53* and *pten* genes, as well as the floxed STOP-cassette inhibiting the expression of the Luc-SIY fusion protein, are deleted using a non-replicative adenovirus. This adenovirus expresses the Cre recombinase under the control of a CMV promoter.

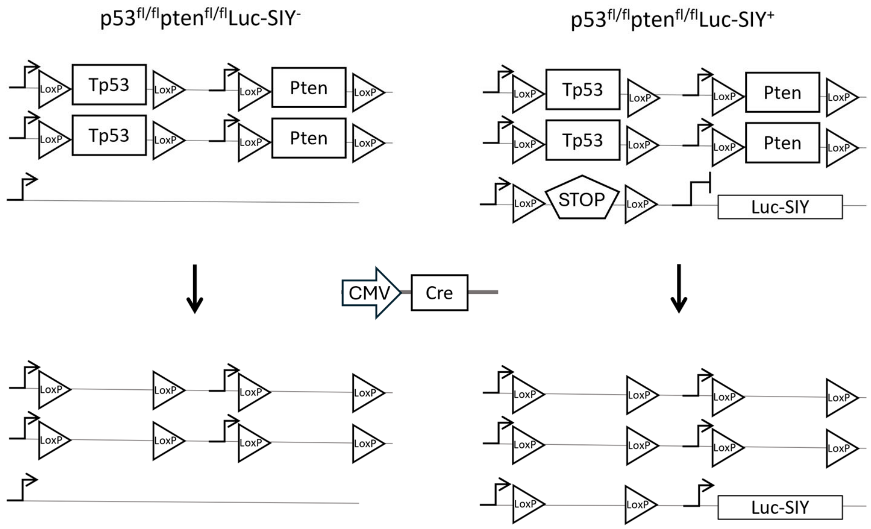

Supplement: Supplementary file 1 [file cancers-16-02245-s001.zip › cancers-3039483-supplementary.pdf]
